# Factors that contribute to physician burnout, interventions to prevent and mitigate burnout and knowledge gaps—a Delphi study

**DOI:** 10.3389/fpubh.2025.1540214

**Published:** 2025-05-21

**Authors:** Catarina Simões, Inês Fronteira, Ana Rita Pedro

**Affiliations:** ^1^NOVA National School of Public Health, Public Health Research Centre, Universidade NOVA de Lisboa, Lisbon, Portugal; ^2^NOVA National School of Public Health, Public Health Research Centre, Comprehensive Health Research Center, CHRC, REAL, CCAL, NOVA University Lisbon, Lisbon, Portugal; ^3^Global Health and Tropical Medicine, Instituto de Higiene e Medicina Tropical, Universidade Nova de Lisboa, Lisbon, Portugal

**Keywords:** burnout, interventions, mental health, NHS, Delphi

## Abstract

**Introduction:**

Burnout among physicians is a worldwide concern issue, impacting individual well-being and healthcare efficiency and jeopardizing the achievement of Universal Health Coverage. Using the Portuguese National Health Service scenario, we aimed at identifying the factors that contribute to burnout of physicians and interventions that can be helpful in its prevention.

**Methods:**

We used a Delphi panel technique with three rounds of participation with 16 specialists, including physicians, psychologists, academics and hospital administrators.

**Results:**

Organizational variables such system strain, staff overload, and unfavourable working circumstances were pointed as the main contributing factors of burnout. Consensus was reached that courses of action, such as resource allocation, legislative changes, and promotion of a healthier workplace environment can help prevent physician burnout. Non-organizational tactics such as workplace amenities and health literacy initiatives were also considered relevant. Identified knowledge gaps comprised long-term effects of burnout, leadership influence, and environmental repercussions.

**Discussion:**

The study concludes that addressing organizational factors and implementing targeted interventions are crucial for improving physician well-being and aiding healthcare efficiency in Portugal but also in similar contexts.

## Introduction

Burnout is a pressing worldwide issue that became even more evident with the COVID-19 pandemic (1, 2). A systematic review looking at the effects of the COVID-19 pandemic on the health of healthcare workers revealed a prevalence of 46% of burnout, and demonstrated an increasing trend manifesting in a series of physical, emotional, cognitive, and behavioral symptoms (3).

Physicians seem to be particularly affected (4). In 2021, a study conducted in the United States of America (USA) revealed that 62.8% of physicians experienced burnout (versus 38.2% in 2020) (1). The demanding nature of the medical work has raised concerns about the mental health and well-being of physicians (5–7). A study in Lebanon revealed that around 70% of physicians had work-related burnout (8). Other studies revealed that levels of burnout varied across subspecialities with emergency doctors and general practitioners being among the most affected (2, 9, 10). Portugal, a 10 million inhabitants southern Europe country, with a National Health Service (NHS) health system is no exception to this scenario. A study among 9,176 physicians revealed that 66% had high levels of emotional exhaustion, 33% depersonalization and 39% decreased personal accomplishment (11).

Burnout is defined in the ICD-11 as a syndrome resulting from chronic workplace stress that has not been successfully managed. It is characterized by emotional exhaustion (feelings of energy depletion), depersonalization (increased mental distance from one’s job, or feelings of negativism or cynicism related to one’s job), and a reduced sense of personal accomplishment (12).

Recent systematic reviews and meta-analyses have significantly advanced our understanding of the complex factors underpinning burnout. They have emphasized the interconnected relationships among burnout, depression, and anxiety, highlighting the multifactorial etiology of the syndrome, while showing that unfavourable work environments are closely linked to the development of burnout symptoms (13, 14). In support of these conclusions, empirical study conducted in Brazil during the COVID-19 pandemic identified key organizational pressures and shown a significant prevalence of burnout among medical staff in public hospital networks (15). Together, these studies show that a combination of environmental, organizational, and individual variables can lead to burnout.

Burnout syndrome not only lowers the quality of life of the individual, and leads to poor mental health but also has broader implications for the healthcare system, potentially jeopardizing patient care and overall system efficiency (10, 16–19). For instance, in 2019, the attributable cost of physician burnout in the United States was 4.6 billion USD (20). Burnout has been linked to a decrease in patient safety, which has resulted in assistance errors, poor outcomes, patient dissatisfaction, and an increase in patient and family complaints (18). Additionally, burnout can lead to a deterioration of teamwork climate, safety, and job satisfaction, all of which have an adverse effect on patient safety (18, 21, 22). Furthermore, burnout has been linked to a higher risk of infections related to healthcare and a decline in the standard of patient care (18, 23). Therefore, tackling burnout among health workers, particularly physicians, is paramount to improve patient safety and the overall system efficiency.

The need to tackle burnout in physicians and improving their physical and mental health and well-being has been recognized (24, 25). Although there is a developing body of knowledge about contributing factors to the syndrome and the best health management strategies to address it, this knowledge is not yet precisely adapted to the particular difficulties faced by doctors at sub-regional and local level. This study aims to address this gap using a Delphi study. By identifying and consensualising these points, we seek to provide practical insights, suggested by specialists that can contribute to physician well-being and enhance the overall sustainability of the healthcare system.

## Materials and methods

Consensus methods aim to reach broad agreement on a controversial issue by having experts propose solutions to a defined problem based on their experience, within a structured framework (26). The Delphi method is a qualitative approach used to systematically gather key insights from a group of experts by collecting, clarifying, and sharing their experiences through a series of questionnaires. This technique relies on four core features: participant anonymity, structured interaction, controlled feedback, and statistical aggregation of group responses (27, 28).

A Delphi panel was conducted between April and May 2024, with the aim to identify factors contributing to burnout and the most effective strategies to prevent and mitigate this syndrome in Portuguese NHS physicians. Portugal is a South European country that has one of the largest ratio of physician per 1,000 inhabitants and one of the lowest of nurses per 1,000 inhabitants (ratio of nurses to doctors of 1.3) which indicates a health system largely dependent of the first (29). Nevertheless, the country faces a shortage of physicians.

**Figure 1:**
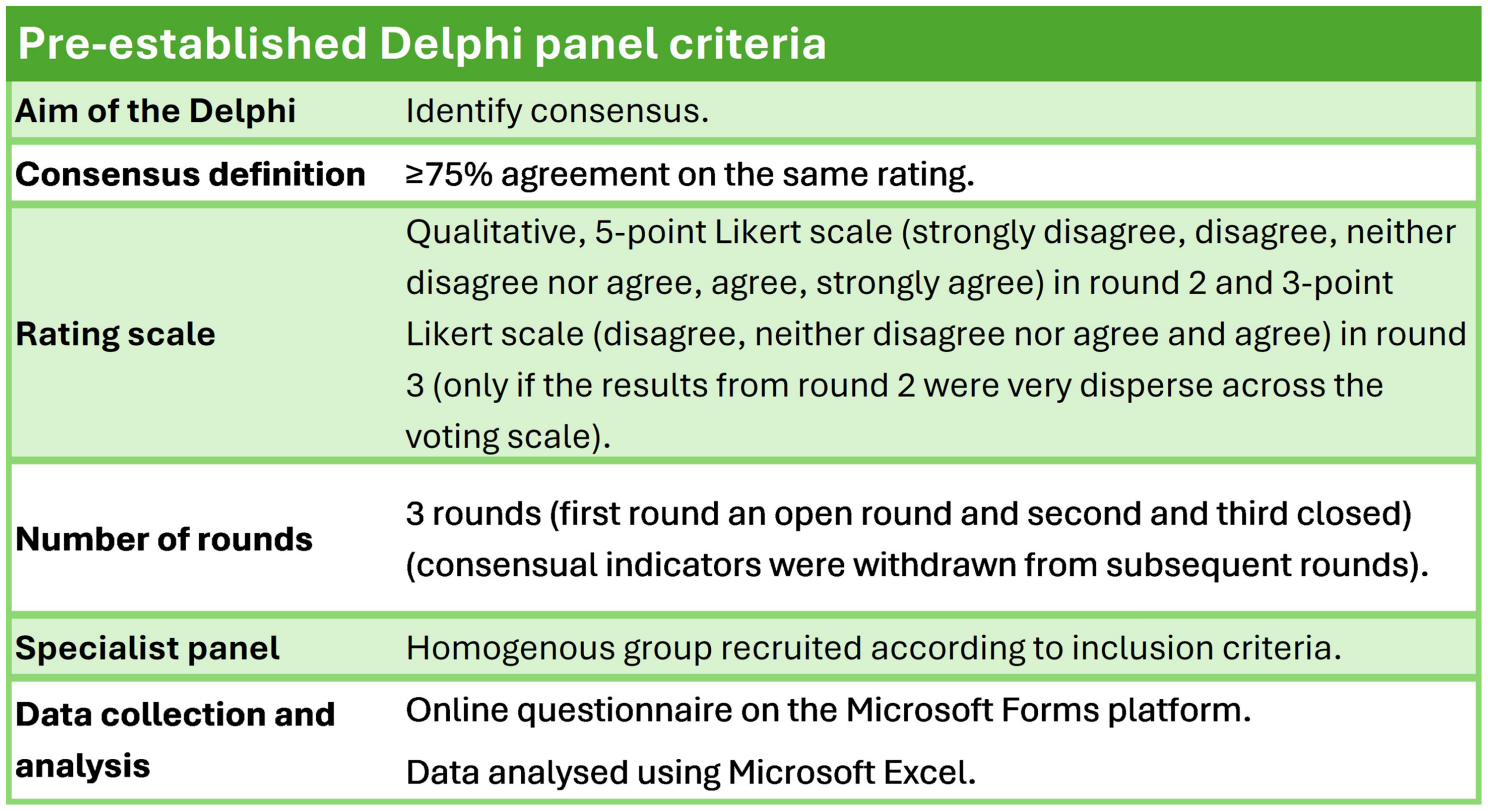
Pre-established methodological criteria for the Delphi panel.

The methodological criteria were all established (Figure 1) before the technique was implemented, ensuring the rigorous design necessary for obtaining reliable and reproducible results.

There were three rounds of questionnaires, based on other healthcare Delphi models and according to the available time. The specialists were invited to participate if they met at least one of the following criteria: have a published article about burnout syndrome in the last 5 years, have at least 10 years of experience as academic in the mental health field or be an hospital administrator.

Invites were sent to 50 Portuguese experts, and 18 of them accepted an invitation via e-mail to respond. Sixteen experts (8 woman and 8 men) completed the 3 rounds of the Delphi panel, their professional backgrounds varied from physicians (6) (3 general practice; 1 public health; 1 occupational health; and 1 psychiatry), psychologists (6), academics (1 occupational health professor) to hospital administrators (3).

As the Delphi technique highly depends on the participation of external experts, one of the study’s greatest concerns was the insufficient number of recruited experts or their dropout during successive rounds. To mitigate this situation, some strategies were implemented: use of an online platform, available anytime and anywhere; adoption of a fully anonymous and confidential methodology; have a maximum of 3 rounds; in round 3, the previous results were presented alongside the indicators for reclassification, encouraging an immediate response; each expert was provided with an individual password to access the questionnaires, enabling the identification of missing responses and allowing for timely reminders.

The decision to invite 50 experts was based on the understanding that response rates in Delphi studies tend to be low, as participation requires a sustained commitment across multiple rounds (30, 31). Prior literature indicates that Delphi panels commonly range from 8 to 20 participants (32). While larger panels can enhance stability by reducing the influence of individual experts, excessively large groups may introduce logistical challenges (33). Given these considerations, we aimed to secure a final panel within this recommended range. Anticipating that not all invited experts would accept, we extended invitations accordingly. The final sample of 16 experts falls within the range suggested in the literature, ensuring a diverse set of perspectives while maintaining methodological rigor within the Portuguese healthcare context.

All the questions were of an obligatory nature so the respondents could not go forward with survey without answering the previous questions first. While answering the forms, the experts could change their answers, however after submission it was impossible to do so.

The first round of the Delphi consisted of three open-ended questions, tackling the contributing factors (“From your point of view, what do you think are the main factors—at an individual, organizational and/or system level—that contribute to burnout among physicians in the Portuguese NHS?), promising tactics/interventions to prevent and mitigate burnout (“What specific health management interventions, inside or outside Portugal, do you consider to be promising in preventing and mitigating burnout among physicians in the Portuguese NHS?), and knowledge gaps (“From your professional point of view, what specific areas or aspects related to physicians’ well-being, burnout or interventions in the area of mental health promotion are still little explored or not well understood?”).

The answers given by the experts that responded to the questionnaire were rounded up, and the bullet points present in them were put as possible answers in the second round of the panel, with the goal of the experts rating them using a 5-point Likert scale (strongly disagree, disagree, neither disagree nor agree, agree, strongly agree).

A consensus was deemed achieved when agreement reached or surpassed 75% for one of the voting options on each indicator. The consensus rate was calculated by dividing the number of respondents who agree on a particular indicator by the total number of respondents who provided answers for that indicator. The result was then multiplied by 100 to convert it into a percentage.

Considering the diverging distribution of responses in the second round – meaning that even with a lower consensus threshold than the one applied (≥75%) agreement would not be possible – it was decided to group the extremes. Specifically, the “strongly disagree” and “disagree” votes were combined, as were the “agree” and “strongly agree” votes. This approach aimed to facilitate the identification of areas of consensus while acknowledging that it may result in some loss of nuance. When consensus was achieved, the indicator was withdrawn from the next rounds.

The statements that did not reach consensus in the second round were sent for a third and final round. For each of the questions, the experts could consult a graph detailing the distribution of votes (percentagewise) on each answer that did not reach the predefined level of agreement in a single voting option. In this final round, the experts were asked to rate the topics based on a 3-point Likert scale (disagree, neither disagree nor agree and agree).

## Results

### Participation and dropout rates

The response rate was 89% since the only existing drop out, between the first and second round, was circa 11%. The final round was answered by 16 specialists.

### Factors that contribute to physician burnout

The main factors that contribute to burnout can be differentiated in individual (if related to the physician) and organizational or system level (if not intrinsic to physician). Out of the 42 factors identified in the answers to the first round, only 3 were strictly related with the physician, namely the difficulty in work life balance, family and financial problems and a predisposition to mental health disorders (genetic or somatic) or undiagnosed mental illness. Of these factors only the difficulty in reconciling professional and personal life reached consensus, with a 75% consensus rate.

Of the 39 organizational or system level factors, only 15 achieved consensus (75% or more level of agreement), making the consensus rate for this type of factors 38%. Among these were high demand, work overload, lack of organizational support and poor management, poor working conditions and remuneration, lack of professional recognition, absence of supporting and reporting mechanisms, non-existence of initiatives to promote professional well-being and the turnover of healthcare professionals. All but one (turnover of healthcare professionals) of the statements reached consensus in the second round.

The factors not intrinsically linked to the physician far outweighed those that were (93% of the statements in the first round). The same pattern was observed in the 16 statements that reached consensus (94% were organizational or system level).

**Figure 2:**
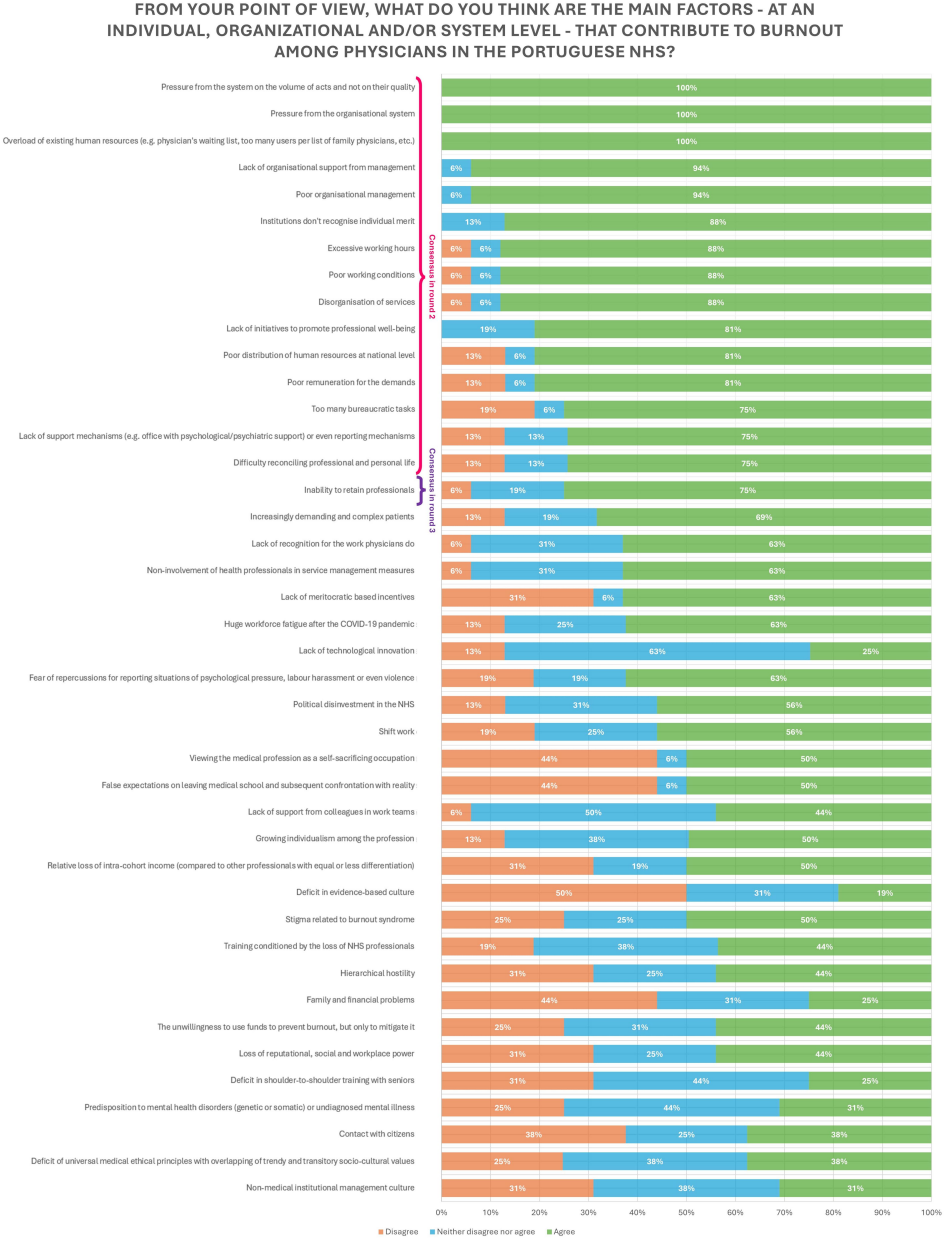
Factors that contribute to burnout among physicians in the Portuguese NHS.

Details on the distribution of the level of agreement/disagreement are presented in Figure 2.

### Interventions to prevent and mitigate burnout in physicians

The interventions to prevent and mitigate burnout can be differentiated according to their focus on management or not. Out of the 36 statements collected from the answers given in the first round, 25 were meant for institutional management, while 11 of them were not.

Of the 25 organizational based interventions, only 14 reached consensuses (consensus rate = 56%). They focused mainly on changing the current policy, increasing the resources allocated to NHS, implementing of report and support mechanisms, improving working conditions (i.e., infrastructure, salaries and distribution of work) and promoting a better work culture by implementing healthier work values and fostering inter-personal relationships between coworkers.

Of the other 11 interventions that did not focus on management, only 7 gained consensuses (consensus rate = 64%). They focused mainly on the creation of supporting services, like gyms and nurseries within the work facilities, implementing a literacy project focused on the theme of burnout aimed at physicians and the managers, as well as increasing the population’s health literacy skills. Promotion and implementation of occupational health measures and of monitoring culture for burnout but also other mental health outcomes (e.g., stress) as well as the implementation of mindfulness and coaching programs were interventions that also achieved consensus.

All the statements that reached consensus did so in the second round.

**Figure 3:**
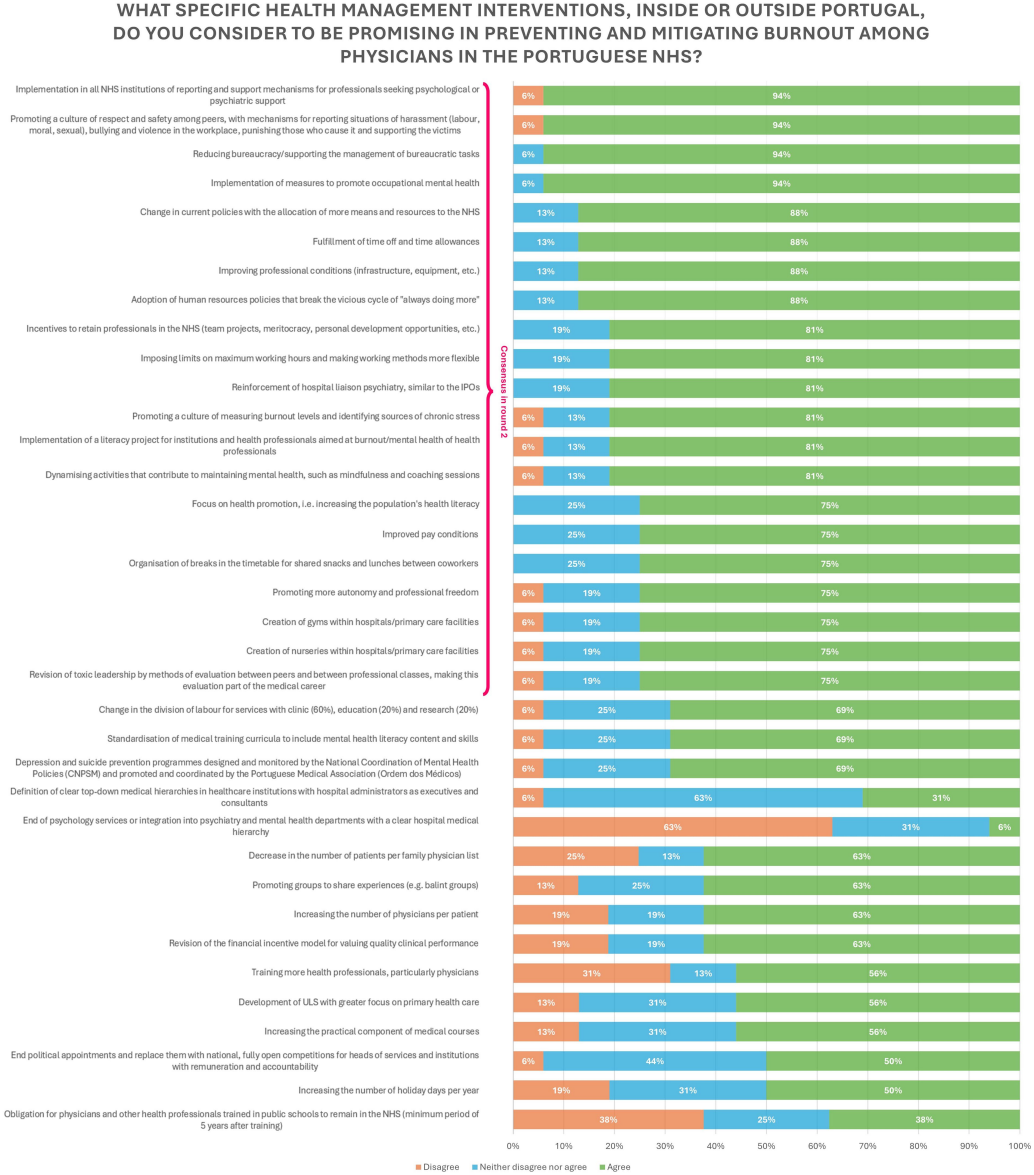
Health management interventions to prevent and mitigate burnout among physicians in the Portuguese NHS.

The interventions targeting the management of the institutions more than doubled those not management related, making up to 69% of the total statements given. The same pattern was observed in the statements that reached consensus (21 in total), where 67% of the statements represented interventions for the organizations. Details on the distribution of the level of agreement/disagreement are presented in Figure 3.

### Knowledge gaps

The knowledge gaps can be classified in physician knowledge gaps and context/environment gaps. Out of the 17 statements collected from the answers given in the first round, 7 of them were strictly related to the physician, while the other 10 were not. All of these 10 reached consensuses, with the higher scores belonging to how work environments can affect clinical outcomes, the influence of leadership on burnout, the long-term effects on healthcare provided by burned out professionals and the diagnosis and monitoring of occupational stress.

Of the 7 knowledge gaps that focused on the individual, 6 gained consensuses (consensus rate = 86%). They focused mainly on connecting burnout with other factors, such as hours spent in the emergency department, for example, or the relationship between burnout and previous experiences of emotional burden. However, the higher percentage of consensus was achieved on the lack of studies addressing mental health issues throughout the various stages of the medical career, on the specificity of the individual responses to adversity and episodes of mental illness and the consequences for the physicians, their families and their patients.

All the statements that reached consensus did so in the second round except for three (individual responses to adversity and episodes of mental illness, relationship between peer and supervision support and burnout, and the relationship between burnout and experiences with previous emotional burden).

The knowledge gaps related with the environment/context outweighed those linked to the physicians, making up 59% of the statements given in the first round. The same pattern was observed in the statements that reached consensus (16 in total), where 63% of the statements were not directly linked with the physicians.

**Figure 4:**
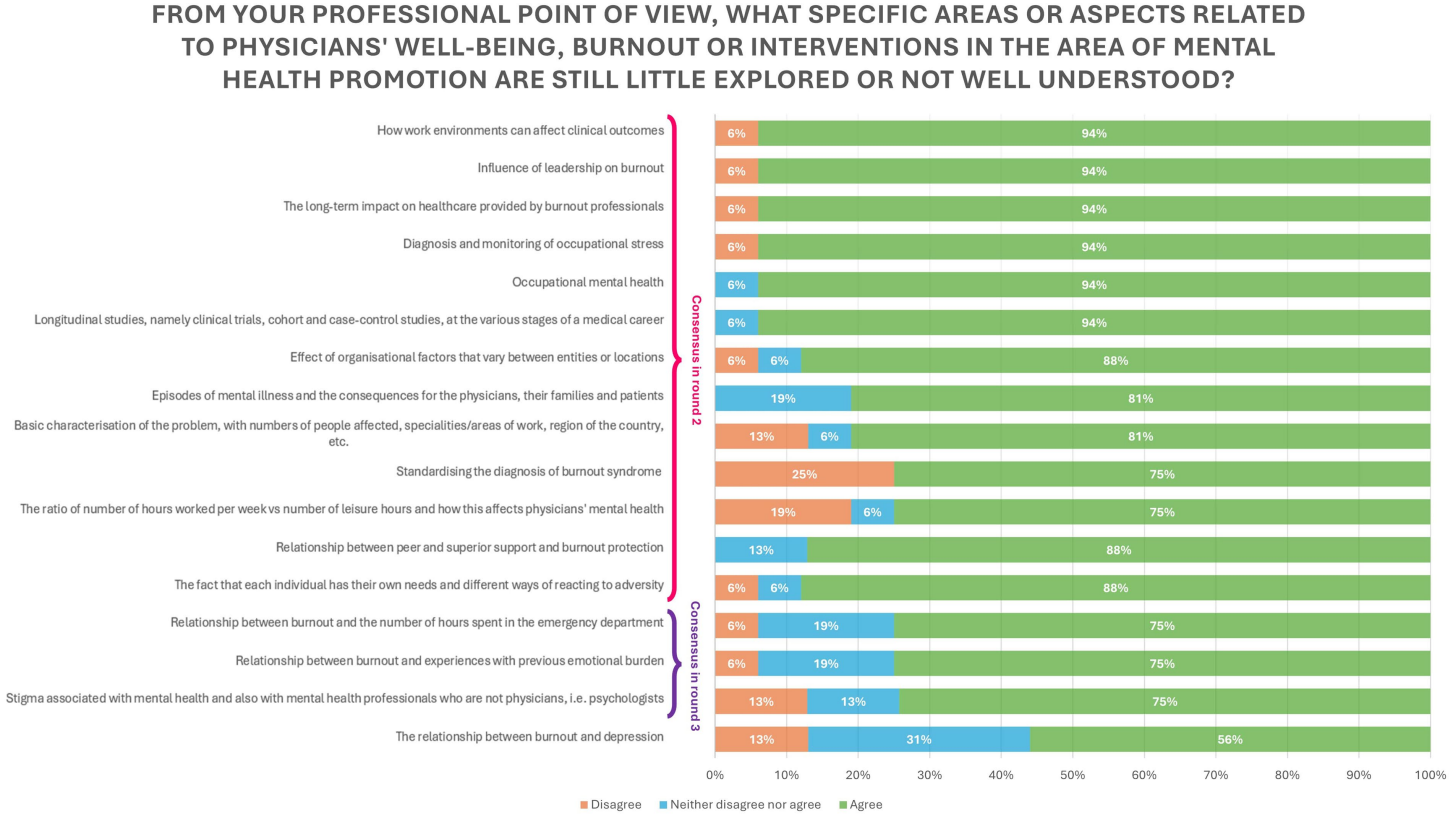
Knowledge gaps in physician’s well-being, burnout or in interventions in the area of mental health.

Details on the distribution of the level of agreement/disagreement are presented in Figure 4.

## Discussion

The pervasive issue of physician burnout has garnered increasing attention in recent years, with a growing body of evidence highlighting the critical role that organizational factors play in exacerbating this problem (34). This study, using a Delphi approach, aimed to identify key factors contributing to physician burnout and interventions to prevent the syndrome and mitigate its impact within the Portuguese NHS. The results of this study emphasize the increasingly clear role of organizational factors in contributing to physician burnout. The consensus reached on factors such as system pressure, work overload, poor working conditions, and lack of organizational support indicates that these systemic issues are more significant contributors to burnout than individual factors, emphasizing the impact of workplace environment on burnout.

Individual factors, such as work-life balance, reached consensus in this panel but were significantly outnumbered by organizational factors. This suggests that while individual interventions are important, they are insufficient on their own and must be part of a broader strategy to addresses system related issues. This hypothesis falls in line with emerging evidence that suggests personal resilience tools must be supported by robust organizational strategies to maximize their effectiveness and help prevent burnout (35, 36).

Most interventions that achieved consensus were also organization focused. These included policy changes, increased resource allocation, better working conditions, and fostering a healthier work environment which indicates a strong recognition among experts that improving the organizational environment is essential for mitigating burnout. The effectiveness of such interventions has been supported by prior research, which suggests that systemic changes can lead to significant improvements in employee well-being and job satisfaction (37, 38).

Interventions such as implementing workplace supporting facilities like gyms and nurseries, promoting burnout literacy, and enhancing occupational health also achieved consensus. While not directly related to management, they address important aspects of work-life balance and personal health, further supporting the need for a holistic approach to burnout prevention, as previous literature has also called for (39, 40).

Despite the increasing understanding of physician burnout, there remains a significant gap in knowledge, highlighting the need for further research to fully grasp its complexities and develop effective interventions (41). With the goal of being a starting point for much needed future research in the area, one of the questions of the Delphi panel focused on the knowledge gaps related to physicians’ well-being, burnout or interventions if the area of mental health promotion. The experts identified several knowledge gaps, particularly in understanding the long-term effects of burnout, the influence of leadership on burnout, and the relationship between work environments and clinical outcomes. These answers emphasized the need for comprehensive studies that explore these aspects in greater depth and that can present evidence-based interventions to prevent burnout and support burned out physicians. Future studies should evaluate the effectiveness of targeted interventions and preventive strategies, providing research-supported solutions to improve healthcare system sustainability.

Although the recommendations derived from this study seek to address physician burnout broadly, there are differences in their viability within the existing Portuguese NHS. Certain suggestions, including expanding access to mental health care and encouraging work-life balance, are in line with NHS policies and programs already in place. Others, including more funding for physician well-being initiatives or structural modifications to task allocation, can call for more resources and changes to existing policies. To evaluate how well these suggestions can be implemented in the present healthcare system, more study and policy debates are required.

While the Delphi panel questions focused on understanding burnout at a Portuguese NHS level, some findings may be relevant to other countries with similar public health systems. The Portuguese NHS shares key characteristics to other public health systems around the world, sharing both strengths and challenges (42). Like many national health systems, the Portuguese NHS seeks to ensure that all citizens, regardless of income, have access to basic medical services (42, 43). However, it has the same problems as publicly funded healthcare around the world, such as high demand, a lack of resources, and a staffing deficit. Long waiting times, ineffective administration, and employee burnout are common problems that are similar across public healthcare in other countries (44, 45). Nevertheless, while the results provide valuable insights for other publicly funded healthcare systems facing similar structural challenges, generalizations should be made cautiously, considering that country-specific factors may influence the applicability of these findings.

This study has some limitations. The reliance on the opinion of experts through a Delphi panel may introduce bias, as the findings are based on the subjective views of those who participated in the study (46). Nevertheless, we tried to mitigate this bias by having a variety of experts ranging from academics to physicians currently practising. The decision to group the extremes of the 5-point Likert scale (i.e., joining the “strongly disagree” and the “disagree” voting options, as well as the “agree” and “strongly agree”) after the distribution of votes in the 2nd round was deemed the most viable solution for achieving conclusive results while maintaining their reliability. Also noteworthy, is the fact that the third and final round did not add many consensuses into fruition which can be perceived as a confirmation that the experts felt strongly about the subject and the votes they gave in the second round remained consistent, even after being asked to consider them again, after changing from a 5-point Likert scale to a 3-point one.

## Conclusion

This study identified significant organizational factors contributing to physician burnout and interventions to prevent and mitigate the syndrome. In this study, the main contributing factors of burnout include inadequate organizational support, system pressure, personnel overload, unfavourable working circumstances, and inadequate compensation. Interventions targeting these factors, such as policy changes, increased resource allocation, and promotion of a healthier work culture, achieved strong consensus among experts. Non-organizational tactics such as the implementation of workplace amenities and health literacy programs were also considered crucial.

By highlighting the importance of organizational factors over individual ones, this research contributes to our understanding of physician burnout and some of the solutions to tackle it. The findings suggest that systemic changes within healthcare institutions are more crucial than interventions targeting individual resilience. By identifying specific organizational interventions, this study contributes to a more nuanced approach to preventing and mitigating burnout. These results may influence management strategies and policies in healthcare systems dealing with comparable problems.

Burnout in the healthcare industry is a complex problem that primarily calls for systemic approaches while also emphasizing the value of individual-based interventions. Subsequent research may examine the interaction between individual and organizational elements, scrutinizing the ways in which personal characteristics and coping strategies intersect with systemic influences. This all-encompassing method might result in more thorough burnout prevention techniques.

## Data Availability

The raw data supporting the conclusions of this article will be made available by the authors, without undue reservation.
